# Effects of *in vitro* fertilization and intracytoplasmic sperm injection treatment on female patients' perinatal mental health: systematic review and meta-analysis

**DOI:** 10.3389/frph.2025.1668831

**Published:** 2025-09-23

**Authors:** Hana Nemcova, Tereza Blaskova, Anna Horakova, Marie Kuklova, Kristyna Hrdlickova, Antonin Sebela

**Affiliations:** ^1^ National Institute of Mental Health, Centre of Perinatal Mental Health, Klecany, Czechia; ^2^ Department of Psychology, Faculty of Arts, Charles University, Prague, Czechia; ^3^ First Faculty of Medicine, Charles University, Prague, Czechia; ^4^ Department of Demography and Geodemography, Faculty of Science, Charles University, Prague, Czechia; ^5^ Department of Epidemiology, Second Faculty of Medicine, Charles University, Prague, Czechia; ^6^ Third Faculty of Medicine, Charles University, Prague, Czechia

**Keywords:** *in vitro* fertilization, intracytoplasmic sperm injection, perinatal mental health, depression, anxiety

## Abstract

**Introduction:**

*In vitro* fertilization (IVF) and intracytoplasmic sperm injection (ICSI) patients often experience stress, which exacerbates the burden associated with infertility and may lead to an increased risk of mental-health difficulties. In this systematic review and meta-analysis, we examined the effects of IVF or ICSI on female patients’ mental health.

**Methods:**

A database search was conducted in PubMed, Web of Science, and PsychInfo to select relevant studies. Forty-four studies involving 858,966 participants were included in the systematic review. The results of these studies were very heterogeneous and yielded contradictory findings. Two meta-analyses, comprising a total of seven studies, were conducted. The first calculated the standardized mean difference of symptoms of depression between women who became pregnant through IVF and those who conceived spontaneously. In the second, we compared symptoms of anxiety between these two groups.

**Results:**

Five studies examined depressive symptoms and showed no significant difference between the two groups: *SMD* = −.15; 95% *CI* [−.33,.03], *p* = .10. A meta-analysis of six studies on anxiety symptoms revealed significantly higher levels in the IVF groups compared to the controls: *SMD* = .33; 95% CI [.17,.49], *p* < .001.

**Discussion:**

The results suggest that the psychological effects of IVF/ICSI, especially with respect to anxiety, require attention and support from healthcare providers, although the effect size is small. Further studies with adequate sample sizes, including women with both successful and unsuccessful treatment, and adequately controlling for important confounders are needed to fully understand the effects of IVF/ICSI on mental health.

**Systematic Review Registration:**

PROSPERO (CRD42023461472).

## Highlights

• Higher levels of anxiety symptoms in the third trimester were found in women after IVF when compared to spontaneously pregnant women, although the effect size is rather small.
• No clear conclusion in terms of effects of IVF or ICSI on other of women's perinatal mental health outcomes could be made.
• Major limitation is omitting women whose treatment was unsuccessful.

## Introduction

Global estimates indicate that around one in six people will deal with fertility problems at some point during their lifetimes (1). Furthermore, the prevalence of both male and female infertility is rising by over 1% per year (2), which has led to a dramatic increase in the use of assisted reproductive technologies (ART) over the past three decades (3).

The Centers for Disease Control and Prevention reports that over 230,000 patients underwent ART treatment which resulted in approximately 92,000 of over 3.6 million live births in the United States in 2021 (4, 5). In 2019, 3% of all live births in Europe resulted from pregnancies conceived with ART, and in some countries the rate surpassed 6%. *In vitro* fertilization (IVF) and intracytoplasmic sperm injection (ICSI) are of the most frequently used ART methods (3).

IVF is often perceived as stressful (6), adding to the psychological strain inherently associated with infertility (7–9). Furthermore, in conventional IVF protocols, hormonal ovarian stimulation is used to enhance the production of oocytes (10), and this stimulation can have adverse effects on patients’ mood (11). These effects of ART on mental health may be persistent or lead to further difficulties, even when it results in conception (12).

Moreover, becoming pregnant itself entails significant changes in parents’ lives; even if the pregnancy is desired, this period of life is associated with increased proneness to developing mental health difficulties (13, 14). In the general population, around 15% of pregnant (15) and 14% postpartum (16) women suffer from major depression. The prevalence of any clinically diagnosable anxiety disorder in either pregnancy or postpartum is estimated at 15% (17). These difficulties can contribute to adverse birth outcomes (18), act as risk factors for subsequent mental-health problems (19, 20), impair bonding with the child (21), and result in health and developmental problems in the child (22, 23).

To prevent or mitigate these adverse outcomes, it is essential to understand the long-term effect of IVF on pregnant women's mental health. To our knowledge, the most recent meta-analysis regarding effects of ART on depressive symptoms was published by Chen et al. (24). Its focus, however, was relatively broad, as it included studies with a variety of designs which concerned any ART and measured depressive symptoms that occurred at any time point during the perinatal period. In their recent systematic review on a similar topic, Capuzzi et al. (25) highlighted the need for a meta-analysis to strengthen evidence and resolve their contradictory findings. To our knowledge, no meta-analysis has been published concerning the effects of ART on the symptoms of anxiety. In the present systematic review, we aimed to summarize the empirical evidence of the effects of IVF and ICSI on women's perinatal mental health. In the meta-analytical portion of the study, we assessed the effect of IVF on symptoms of depression and anxiety in the third trimester of pregnancy. This targeted analysis aimed to provide a more refined understanding of the relationship between IVF and perinatal mental health during this critical stage.

## Methods

### Search strategy

The systematic review and meta-analysis were conducted according to The Preferred Reporting Items for Systematic reviews and Meta-Analyses (PRISMA) guidelines (26). A protocol was registered in the PROSPERO register (ID: CRD42023461472). We intended primarily to conduct a systematic review concerning the effects of IVF and ICSI on perinatal mental-health problems with the option of conducting meta-analysis focusing on a more detailed topic if the relevant data were available.

We performed a database search of studies published through September 2023 in PubMed, Web of Science, and PsychInfo. The search terms were: (“IVF” OR “*in vitro* fertilization” OR “*in vitro* fertilisation” OR “ICSI” OR “intracytoplasmic sperm injection”) AND (“mental health” OR “mental disorders” OR “distress” OR “anxi*” OR “depress*” OR “psychopathology” OR “emotional distress”). For the selection and data extraction of studies, the Covidence tool (27) was used.

### Study selection

The abstracts and titles of the selected studies were screened, and the full texts of studies included in the first step were reviewed independently by two reviewers (HN and TB). In the case of conflict, a third reviewer (AH) decided on the study's inclusion.

The inclusion criteria were: (1) studies concerning female patients 18 years or older; (2) the intervention used was IVF or ICSI; (3) the outcome measures were symptoms of distress or diagnoses or symptoms of mental disorder; (4) the assessment of outcome measures occurred between the start of the treatment and one year postpartum. We included all studies published since 1978, when IVF was first introduced (28). The exclusion criteria were: (1) studies not in English; (2) animal studies; (3) the timing of assessment of outcome was not specified; (4) qualitative studies, reviews, or meta-analyses; (5) studies using solely native IVF cycle.

For the purposes of the meta-analysis, the included studies were screened for the most frequent study design used with participants of similar gestational age or time point postpartum. Consequently, the meta-analysis included studies that used self-reporting scales to assess symptoms of depression and/or state of anxiety. Participants were women in the third trimester of pregnancy who had undergone IVF and were compared to spontaneously pregnant women.

### Data extraction

From the chosen studies, the following data were extracted independently by HN and TB using Covidence software (27): name of the lead author; year of publication; country in which the study was conducted; year of data collection; aim; study design; population description; inclusion criteria; exclusion criteria; use of a control group; characteristics of the control group; total number of participants; intervention used; outcome variable; timing of outcome assessment; and findings. For the purposes of statistical analyses, sample sizes, means and standard deviations of scores on scales measuring symptoms of depression and anxiety for both the IVF and control groups were compiled. Afterward, the data extracted by HN and TB were discussed, and a final consensus was reached.

### Quality assessment

To assess the quality of the studies, the Risk of Bias in Non-randomized Studies of Intervention [ROBINS-I; (29)] was used. This tool is suitable for studies comparing the effects of interventions between groups without employing randomization. The bias of each study was evaluated as *low, moderate, serious*, or *critical* in seven domains.

### Statistical analysis

To perform the statistical analysis, Stata 17.0 software was used. A Q-test was used to estimate the heterogeneity of included studies. The standardized mean difference between the IVF and control groups was assessed using a random-effects models (30) separately for symptoms of anxiety and symptoms of depression. The risk of publication bias was assessed using a fail-safe N test, Begg and Mazumdar rank correlation test, and Egger's regression test. A funnel plot was not used, as we included fewer than 10 studies (31).

## Results

### Literature search

**Figure 1:**
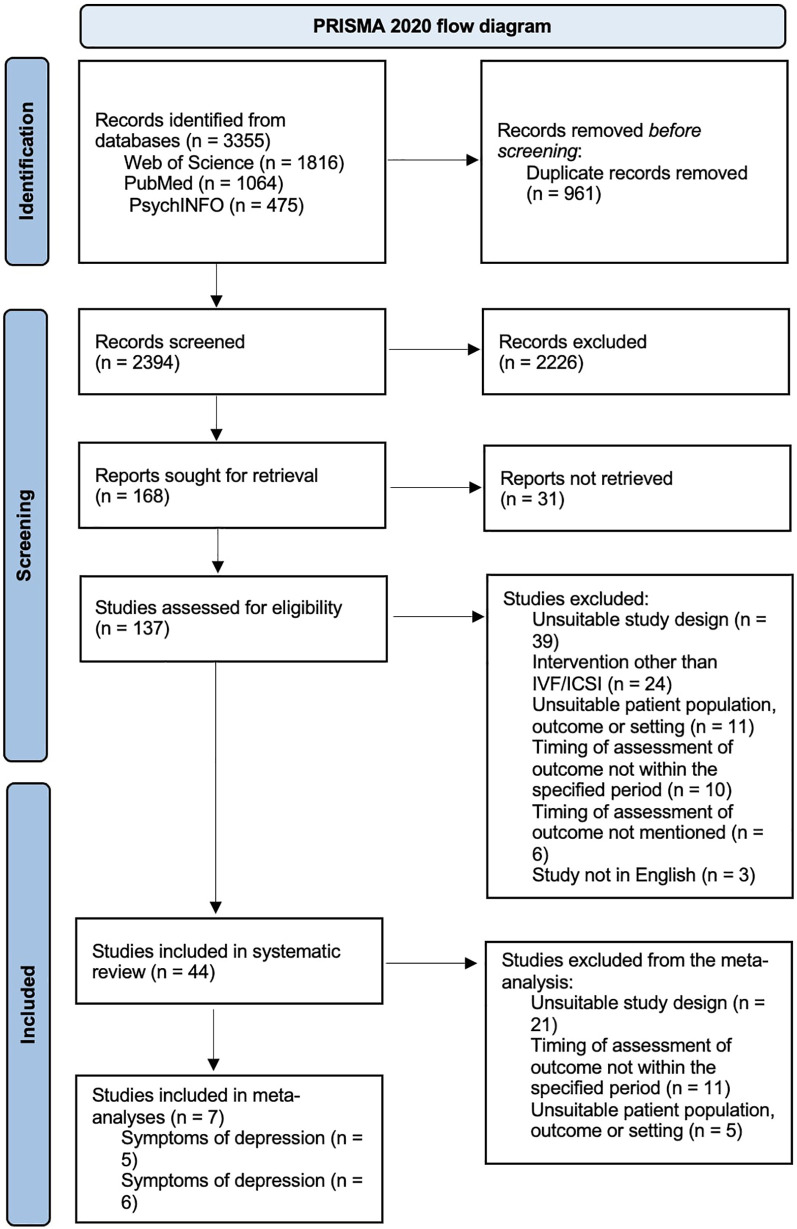
Flow diagram of literature search.

As indicated in Figure 1, the database search yielded a total of 3,355 records: 1,816 from Web of Science, 1,064 from PubMed, and 475 from PsychInfo. After excluding 961 duplicate studies, the title and abstract screening involved 2,394 records, of which 2,226 were excluded and 31 did not have their full text available. A full-text assessment was conducted on 137 studies, of which 44 studies were included in the systematic review. Seven of these 44 studies were included in the meta-analyses: five in the meta-analysis on symptoms of depression and six in the meta-analysis on symptoms of anxiety.

## Systematic review

### Study characteristics

Table 1 presents the characteristics of the included studies. They involved 858,966 participants and sample sizes ranging from 37 to 786,064. Twenty-one studies were conducted in Europe, 14 in Asia, five in North America, and two in Australia; two studies did not provide location information. The most reported outcome variable was symptoms or diagnosis of depression (28 studies). Furthermore, symptoms or diagnosis of anxiety (27 studies), stress (5 studies), any mental health disorder or problems (4 studies), and psychotic disorders (1 study) were also assessed.

**Table 1:** Characteristics of studies included in the systematic review.

Study	State	Study design	Number of participants	Aim	Intervention	Outcome variable (assessment method)	Timing	Findings
(40)	**Greece**	**Cohort study**	**95 (19 pregnancies after IVF, 76 spontaneous pregnancies)**	**To examine the possible association between conception via *in vitro* fertilization (IVF) and anxiety or depression during the third pregnancy trimester in** **the Greek population during the years of financial crisis.**	**IVF**	**Anxiety (HAM-A, 0–17 mild anxiety, 18–24 mild to moderate anxiety, 25–30 moderate to severe anxiety, above 30 severe anxiety), depression (BDI, 17–20 borderline clinical depression, 21–30 moderate depression, 31–40 severe depression, above 40 extreme depression)**	**30th–32nd gestational week**	**The IVF group had a higher rate of anxiety (18.8%) and a lower rate of depression (9.4%) than the spontaneous conception group (13.5% and 13.5%, respectively), but the differences were not statistically significant before and after propensity score matching. There were also no statistically significant differences between the HAM-A and BDI total scores of the two groups compared as far as HAM-A and BDI are concerned. After matching the two groups with propensity scores for 10 variables, the tendency of higher anxiety and lower depression in the IVF group remained, but the differences were still non-significant.**
(53)	India	Longitudinal study	137	To evaluate the state and trait anxiety and to measure the perceived stress levels in women undergoing the treatment of IVF at three stages.	IVF + ICSI	Anxiety (STAI), perceived stress (PSS)	On the day they start their treatment (T1), on the day of embryo transfer (T2) and 10 days after the embryo transfer (T3)	The overt anxiety was highest at T3 level (mean = 45.77) followed by T1 level (mean = 44.23) and T2 level (mean = 43.04). Perceived stress was elevated at T1 level (mean = 17.93) followed by T3 level (mean = 17.28) and T2 level (mean = 16.72). The results of ANOVA showed a significant difference in anxiety among all the three levels (*P* = 0.036), but no significant difference was found for perceived stress (*P* = 0.169). A significant difference was only seen between the T1 and T2 levels in perceived stress (*P* = 0.052). In state anxiety, a significant difference was observed only between T2 and T3 levels (*P* = 0.02).
Cozzolino et al. (57)	Italy	Cohort study	245 (105 group A – spontaneous pregnancies, 119 group B – homologous IVF, 21 group C – heterologous IVF)	To explore possible differences in the psychological condition of women who had conceived spontaneously versus women who had conceived with homologous and heterologous fertilization within one year after delivery.	IVF	Depression (EPDS; cutoff 10)	On the day of discharge after childbirth (T1), 1 month (T2), 3 months (T3), 6 months (T4), and 1 year after childbirth (T5)	In the first year postpartum the incidence of psychological disorders was different exclusively at discharge from hospital (24.8% A vs. 38.7 B vs. 19% C) [*P* < .05] and one year after childbirth (13.3% A vs. 3.4% B vs. 4.8% C) [*P* < .05]. Our results suggest a high correlation between PPD and pregnancies resulting from homologous IVF at the time of discharge, whereas there is a higher chance that spontaneous pregnancies develop postpartum depression one year after delivery.
Dayan et al. (72)	Canada	Cohort study	786,064 (688,970 spontaneous pregnancies, 78,283 spontaneous pregnancies after subfertility without infertility treatment, 9,178 pregnancies after ovulation induction or insemination, 9,633 pregnancies after IVF or ICSI	To assess the incidence of mental illness within 1 year postpartum in relation to subfertility and type of infertility treatment and to evaluate associations between subfertility or infertility treatment and postpartum mental illness.	IVF + ICSI	Mood or anxiety disorders, psychotic disorder, substance use disorder, self-harm event or other conditions, such as an eating disorder or an obsessive-compulsive disorder (diagnosis)	0–365 days after giving birth	Postpartum individuals who conceived by invasive infertility treatment had a lower crude absolute risk, but higher adjusted RR of the mental illness composite outcome compared with those who conceived spontaneously (60.4 per 1000 births, adjusted RR 1.14, 95% CI 1.05–1.24). 3.5 per 1000 conceived by IVF had a mood or anxiety diagnosis.
Gabnai-Nagy et al. (58)	Hungary	Cohort study	87	The aim of the study was to explore to what extent positive and negative affectivity and a predisposition to depression and anxiety appear in infertile couples during *in vitro* fertilization (IVF) treatment. We also aimed to explore how the emotional state of couples changed during IVF in relation to treatment outcomes.	IVF	Depression (BDI), anxiety (STAI)	At the beginning of treatment (before hormonal stimulation; T1), before embryo transfer (T2), and before taking a pregnancy test (T3)	IVF women experienced significantly lower level of state anxiety than the female controls (general population norms) at T1 (T1: t (51] = −3.858, *p* < 0.01) and T3 (T3: t [48] = −3.655, *p* < 0.05). Depression M (SD) norms: 8.62 (11.74) IVF T1 6.41 (7.16), *p* < 0.01 IVF T2 5.62 (6.19), *p* < 0.001 IVF T3 6.67 (6.54), *p* < 0.01.
Gambadauro et al. (41)	**Sweden**	**Longitudinal study**	**3,283 (167 pregnancies after IVF, 3,116 spontaneous pregnancies)**	**To evaluate whether conception by IVF is associated with maternal depressive symptoms, both during pregnancy and postpartum, in a large prospective cohort of pregnant women receiving obstetrical care at a Swedish university hospital.**	**IVF** **+** **ICSI**	**Depression (EPDS cutoff 12 and as a continuous variable)**	**17 (T1) and 32 gestational weeks (T2) and 6 weeks (T3) and 6 months postpartum (T4)**	**The prevalence of significant maternal depressive symptoms during pregnancy and postpartum was not significantly different between IVF and spontaneous pregnancies. Similarly, no differences were seen when comparing EPDS scores by means of the Mann–Whitney U test. The logistic regression analyses showed that the mode of conception was not associated with significant depressive symptoms (EPDS >=12) at any of the considered time points during pregnancy and postpartum, even when adjusting for age, BMI, parity, education, depression history, and SLEs. Prevalence of EPDS >= 12 gestational week 17 in IVF 9% (15 women) (spontaneous 13%, 404 women, *p*** **=** **.131), gestational week 32 in IVF 11.5% (spontaneous 12.4%, 353 women, *p*** **=** **.746), postpartum week 6 IVF 14.6%, 22 women (spontaneous13.7%, 354 women, *p*** **=** **.770), postpartum 6 months IVF 9.1%, 13 women (spontaneous 12%, 275 women, *p*** **=** **.291)**
García-Blanco et al. (59)	**Spain**	**Cohort study**	**243 (60 pregnancies after IVF, 183 spontaneous pregnancies)**	**To explore if IVF affects the course of anxiety and depressive symptoms as well as physiological stress from pregnancy to postpartum period.**	**IVF**	**Depression (BDI/SF cutoff 4 and as a continuous variable), anxiety (STAI-S cutoff 19 and as a continuous variable)**	**Third trimester of pregnancy (T1), 48 h after birth (T2) and 3 months after birth (T3)**	**Relative to natural conception mothers, IVF mothers had higher STAI-S scores at T1(*P*** **=** **0.016, odds ratio (OR)** **=** **2.46), and this difference remained steady from T1 to T2 (*P*** **=** **0.37, OR** **=** **0.70) and from T2 to T3 (*P*** **=** **0.36, OR** **=** **0.69). In the case of depressive symptoms, the IVF group obtained lower BDI/SF scores at T1 (*P*** **<** **0.001, OR** **=** **0.192). This difference was apparently reduced from T1 to T2 (*P*** **=** **0.072, OR** **=** **2.21) and remained constant from T2 to T3 (*P*** **=** **0.107, OR** **=** **2.09). It is important to note that whereas the mean BDI/SF score was not clinically significant for any group (it was lower than the cut-off 4), the mean STAI-S score of the IVF group at T1 was so (it was higher than the cut-off 19).**
Globevnik Velikonja et al. (60)	Slovenia	Cross-sectional study	112 (49 pregnancies after IVF, 63 spontaneous pregnancies)	To determine whether pregnant women conceiving IVF differ from those conceiving spontaneously in terms of psychological well-being and the quality of life.	IVF	Depression (BDI), anxiety (SAS)	5th to 26th weeks of pregnancy	No significant differences between groups in depression and anxiety.
Harf-Kashdaei & Kaitz (61)	**Israel**	**Cross-sectional study**	**60 (30 pregnancies after IVF, 30 spontaneous pregnancies)**	**To describe the affective experience of a sample of pregnant women who conceived by IVF and to examine its relation to aspects of the women's history of infertility.**	**IVF**	**Depression (EPDS), anxiety (STAI)**	**On average 30th – 31st week of pregnancy**	**Differences between groups were not significant (Trait anxiety F** **=** **1.21, *p*** **>** **.20, effect size.03; State anxiety F** **=** **1.7 *p*** **>** **.20, effect size.04, Depression F** **=** **0.20, *p*** **>** **.20, effect size.003).**
Harlow et al. (54)	United Kingdom	Longitudinal study	75 (25 women undergoing unstimulated IVF cycle, 26 women undergoing stimulated IVF cycle, 24 women undergoing laparoscopy for sterilization)	To examine whether there is a relationship between hormonal markers and STAI scores.	IVF	Anxiety (STAI)	Initial consultation (T1), early follicular phase between days 2 and 4 (T2), pre-operative sample on the day prior to surgery in the control group, or on the day the dominant follicle reached 15 mm diameter in the unstimulated IVF group, or on the day of human chorionic gonadotrophin injection in the stimulated IVF group (T3)	State anxiety was significantly higher (*P* < 0.05) in the stimulated compared with the unstimulated group at all three time-points (38 versus 34, 40 versus 35 and 49 versus 33). In the stimulated group, the levels of anxiety in T2 were significantly higher than in the control group. State anxiety also increased significantly (*P* < 0.01) during treatment in the stimulated IVF group (38 versus 40 versus 49). State anxiety was also lowest at the baseline point (37) and was significantly higher (*P* < 0.05) at the pre-operative point (40.5). The score fell slightly in the luteal phase (39), although this was not significant.
Hashemieh et al. (46)	Iran	Cross-sectional study	100	To determine the anxiety level in Iranian ARTs pregnant women and factors influencing it including cause of infertility, numbers of treatment failure, different types of ART treatments, infertility.	IVF + ICSI	Anxiety (BAI [without anxiety (<9), mild (10 to 18), moderate (19 to 29), and sever (30 to 63)]	Gestational age from 8 to 42 weeks	Study results showed that 32.5% of IVF subjects were anxious (moderate and sever levels in total). 41.9% of IVF subject had no anxiety, 25.6% of IVF subject had mild anxiety, 20.9% of IVF subject had moderate anxiety and 11.6% of IVF subject had sever anxiety. 75% of ICSI subjects had mild anxiety but the sample is very small containing only 4 women.
Jongbloed-Pereboom et al. (73)	Netherlands	Cohort study	196 (113 pregnancies after IVF, 83 subfertile women who conceived spontaneously)	To investigate whether IVF/ICSI itself and factors related to IVF/ICSI affect mental health and anxiety in women and men 1 year after childbirth.	IVF + ICSI	Common mental health problems (GHQ cutoff above the 80th percentile)	1 year after childbirth	The IVF/ICSI and NC group showed similar GHQ scores (not significantly different). When entered into the multivariate regression analysis only the association with the number of ART treatment cycles remained statistically significant [OR 0.79, 95% CI (0.64– 0.97). Univariate analysis indicated that clinically relevant female GHQ scores were associated with a maternal cause of subfertility [x^2^ (1) = 4.975, *P* < 0.05] and less significantly with the number of ART treatment cycles (U = 2839.50, z = 21.67, *P* < 0.1). When adjusting for confounding factors, only the association between clinically relevant female GHQ scores and maternal cause of subfertility remained statistically significant [OR 0.33, 95% CI (0.13–0.83)].
Kato et al. (32)	Japan	Cohort study	513	To examine mental health and health-related quality of life among women at early stages of treatment.	IVF + ICSI	Depression (QIDS, five-category variable: none, mild, moderate, severe, and very severe), anxiety (STAI, using the total scores for state and trait, each was categorized into 5 levels where 1 represented the lowest anxiety level and 5 was the highest; we defined categories 4 and 5 as high anxiety)	Early stages of treatment	Mild depressive symptoms or worse, assessed with QIDS, were observed among 54% of participants. Mean score for State-Trait Anxiety Inventory was 52 with a standard deviation of 11 for the state, and 39% were categorized as high anxiety.
Klock & Greenfeld (62)	**USA**	**Longitudinal study**	**114 (74 pregnancies after IVF, 40 spontaneously conceived pregnancies)**	**To assess the psychological status (marital adjustment, self-esteem, and levels of depression and anxiety) of IVF patients compared to normal fertile women during the first and third trimesters of pregnancy and at postpartum**	**IVF**	**Depression (BDI), anxiety (STAI)**	**12nd and 28th gestational week**	**There were no significant differences between groups on any of the outcome measures assessing psychological status at the two assessment times. Within-group changes over time indicated that IVF women, not controls, showed a decrease in anxiety during pregnancy: the IVF group also had a significant decrease in state anxiety over time (T1** **=** **35.2, T2** **=** **32.0, t** **=** **2.62, *P*** **<** **.01).**
Kong et al. (33)	China	Cohort study	567 (260 undergoing IVF, 277 women in childbearing age)	To quantitatively analyze the psychosocial characteristics of IVF-ET couples and normal couples, and to identify the influencing factors of psychological characteristics and pregnancy outcomes.	IVF	Depression (SDS, 53–62 points mild depression, 63–72 points moderate depression, and >72 points severe depression), anxiety (SAS, 50–59 points mild anxiety, 60–69 points moderate anxiety, and >70 points severe anxiety)	During IVF	The SAS score of female patients receiving IVF-ET was 42.72 ± 7.60, and the SDS score was 47.66 ± 10.06 which were all significantly higher compared with those in the control group (all *P* < 0.01). The anxiety rate was calculated as 14.2%, depression rate of 30.8%. Anxiety and depression coexisted in 23 patients with a rate of anxiety complicated with depression of 8.85%.
Lee et al. (43)	Taiwan	Cross-sectional study	60	To evaluate factors associated with postpartum depression in women who received IVF treatment	IVF	Depression (BDI, 0–13 minimal depression, 14–19 mild depression, 20–28 moderate depression, 29–63 severe depression)	Within 2nd month postpartum	The prevalence of postpartum depression was 25%, including mild (16.7%), moderate (6.7%), and severe (1.7%).
Li et al. (44)	Taiwan	Cross-sectional study	180	To examine the prevalence of postpartum depressive symptoms among women who conceived while receiving infertility treatment and to explore the associated factors.	IVF	Depression (EPDS, cutoff 10)	2–6 months postpartum	The prevalence of postpartum depressive symptoms was 46.8%
Lin et al. (70)	Taiwan	Longitudinal study	100	To investigate the comparison of (1) somatic symptoms, sleep disturbance and psychological distress; (2) the factors associated with perceived psychological distress and (3) sleep quality and its seven elements in OPU and IVF–ET women.	IVF	Distress (BSRS-5, cutoff 6)	At the time of oocyte pick up (T1) and embryo transfer (T2)	The average of their BSRS-5 scores was 4.01 (SD = 3 63), ranged from 0 to 15, during OPU and 7.48 (SD = 4 50), ranged 0–18, during IVF–ET. Using a cut-off point (score ≥6) for the analysis of psychological distress, 29 OPU participants experienced greater than mild psychological distress, compared with that in 61 IVF–ET participants. Both groups showed a significant difference (*p* < 0 001).
Liu et al. (34)	China	Longitudinal study	247	To examine the differences in anxiety and depression between infertile Chinese couples in diverse stages IVF-ET and their relationship with the IVF-ET outcomes	IVF	Depression (SDS, cutoff 50), anxiety (SAS, cutoff 50)	On the day they started their treatment (T1), the day human chorionic gonadotropin was administered (T2), and 4 days after the embryo transfer (T3)	The incidence of anxiety in women in the T1, T2, and T3 stages was 29.96%, 44.94%, and 17.81%, respectively. The anxiety scores of women were 46.14 ± 8.37, 50.83 ± 8.50, and 44.09 ± 8.17, respectively. The anxiety score in stage T2 was the highest in women, and the depression score of women in stage T1 was the highest. The incidence of depression in women receiving IVF-ET was 15.79%, 9.31%, and 6.88% in the three stages, respectively. The depression score of women in the T1 stage was also the highest, and it was significantly higher than in the T2 and T3 stages. The incidence of anxiety and depression was not significantly different in diverse stages.
Lukse & Vacc (35)	USA	Longitudinal study	50	To identify the levels of grief and depression and the coping mechanisms of women with infertility problems who participated in IVF or ovulation-induction medication	IVF	Depression (DACL, cutoff 13)	4–6 weeks prior to IVF treatment (T1) and within 4 weeks from the date of their anticipated pregnancy (T2)	Thirty-six % scored above 13 on the Depression Adjective Checklist pretest, 40% scored above 13 on the Depression Adjective Checklist posttest. Pretest and post-test means and standard deviations on the Depression Adjective Checklist were not statistically significant for the IVF group (t [49] = 1.29), *p* = .200
Mahajan et al. (55)	India	Longitudinal study	74	To identify pattern of change in average positive affect, negative affect, and state anxiety across three biological end points of an IVF/intracytoplasmic sperm injection procedure and to examine whether the pattern varied across sociodemographic and biomedical subgroups.	IVF + ICSI	Anxiety (STAI)	At baseline (T1), maximum 24–36 hours before OPU (T2), and maximum 24–36 hours before embryo transfer (T3)	The mean state anxiety at baseline (T1) was significantly lower than the average state anxiety at T2 and T3 (*P* = .02). The *post-hoc* pairwise comparison between state anxiety at T2 and state anxiety at T3 showed that the mean state anxiety at T2 was not significantly lower than state anxiety at T3. ANOVA of the 3 time points (F = 3.80, *p* = .02).
Massarotti et al. (48)	Italy	Longitudinal study	89	To evaluate how quality of life, anxiety and depression in infertile women are impacted by infertility treatments, comparing them before planning the treatment and during the IVF cycle. Moreover, secondary objective of the study is to find, if any, subgroups of infertile women that may be more at risk of experiencing distress and subsequently could be a target for psychological counseling.	IVF	Depression, anxiety (HADS, cutoff 8)	Before their first cycle of infertility treatment (T1) and at the end of the ovarian stimulation for *in vitro* fertilization but before the egg retrieval (T2)	Anxiety levels (pretreatment 6.84 ± 3.62 versus during treatment 5.74 ± 4.02, *p* = .004) were lower during the treatment than before, with a mean of differences: –3.85 for anxiety. Depression levels were instead equally low in the two fillings (2.97 ± 2.51 versus 2.77 ± 2.06, *p* > .05).
McMahon et al. (67)	**Australia**	**Cohort study**	**132 (70 pregnancies after IVF, 62 spontaneously conceived pregnancies)**	**To compare 70 couples who had conceived by IVF with 63 matched controls for the prevalence of anxiety and quality of attachment to the baby during pregnancy.**	**IVF**	**Anxiety (STAI)**	**28th–33rd gestational week**	**The multivariate test showed a tendency for the IVF mothers to differ from the control group mothers on state and trait anxiety (F** **=** **2.57, df** **=** **1123, *P*** **=** **0.080). Univariate tests indicated that the effect was the result of somewhat elevated scores on state anxiety for IVF mothers (F** **=** **3.36, df** **=** **1123, *P*** **=** **0.07).**
McMahon et al. (63)	Australia	Cohort study	127 (65 women undergoing IVF, 62 women with no history of infertility)	To examine psychological adjustment to early motherhood at 4 months postpartum in mothers who conceived by IVF-ET.	IVF	Depression (EPDS), anxiety (STAI)	30th gestational week (T1), 4 months postpartum (T2) and 12 months postpartum	The multivariate tests showed no differences between the complete IVF-ET group or either of the treatment cycle subgroups and the control group on the mood state measures of anxiety and depression.
Merari et al. (49)	Israel	Longitudinal study	113	To investigate concurrently the psychological and hormonal changes at three critical points of time during the process of IVF treatment, following the initial assessment of pretreatment baselines.	IVF	Depression (DACL), anxiety (STAI)	Before the onset of the hormonal treatment (T1), in the morning of the day of oocyte retrieval, shortly before the actual retrieval (T2), in the morning of the day of embryo transfer (T3), and in the morning of the day when blood samples were taken for pregnancy tests (T4)	State anxiety scores of the women in all phases of the IVF treatment were significantly higher than the population norm, which is 33.8 (T- test comparison between means, *P* < 0.00001 for each phase). Repeated-measures ANOVA revealed a significant effect of the phase of treatment in state anxiety (dr = 3; F = 15.98; *P* < 0.0001). No significant difference was found between C (concieving) and NC (non-concieving) women and the interaction of phase × conception was not significant either. DACL scores of the women in all phases of the IVF treatment, with the exception of T3, where significantly higher (*P* < 0.002, T-test comparisons between means, two-tailed) than the population norm (8.59). Repeated-measures ANOVA revealed a significant effect of the phase of treatment (df = 3; F = 9.84; *P* < 0.0001). No significant difference was found between C and NC women and the phase × conception interaction was not significant either.
Munk-Olsen & Agerbo (74)	Denmark	Cohort study	21,276	To study whether childbirth is associated with psychiatric episodes and whether having a planned pregnancy and subsequently giving birth to a wanted child prevents postpartum psychiatric disorders, using data from a nationwide IVF register.	IVF	Psychiatric disorders (psychiatric inpatient or outpatient treatment, ICD-10 diagnoses)	0–365 days postpartum	The incidence of onset of any type of psychiatric disorder 0 to 90 days postpartum was 11.3 per 1,000 person-years (95% CI = 8.2–15.0) compared with 3.8 (3.4–4.3) among women not giving birth. Across the various models and after adjustment for selected confounders, women subsequently becoming mothers had higher risks of experiencing a psychiatric episodes 0 to 90 days after the delivery, compared with the women who remained childless (for episodes 0–90 days postpartum, crude irr = 2.8 [95% ci = 2.0–4.0] and fully adjusted irr = 2.9 [2.0–4.2). Additionally, IRRs for inpatient versus outpatient treatment 0 to 90 days postpartum were 3.5 (2.1–5.8) and 2.2 (1.3–3.6), respectively. The IRR associated with psychiatric episodes within 0 to 30, 0 to 60, 0 to 182, and 0 to 365 days after delivery were 3.3 (2.0–5.5), 3.3 (2.2–4.8), 1.8 (1.3–2.5), and 1.2 (0.9–1.5), respectively.
Raoul-Duval et al. (45)	France	Cohort study	99 (33 women who gave birth after IVF pregnancy, 33 women after ovulation induction, 33 women after spontaneous conception)	To investigate the outcomes of children conceived by IVF techniques, The specific aim is to assess the possible influence of this form of assisted procreation on child development and mother-child bonding.	IVF	Depression (interview)	Directly postpartum (T1) and 9 months postpartum (T2)	Postpartum: 15% IVF mothers had symptoms of postpartum depression, 21% of the ovulation induction group, 15% general control - differences are not significant. At 9 months postpartum 35% IVF mothers had symptoms of postpartum depression, 23% of the ovulation induction group, 16% general control - differences are not significant.
Reading et al. (64)	NI	Longitudinal study	47 (37 women undergoing IVF, 10 not pregnant women)	To quantify fluctuations in psychological state over the course of IVF treatment and to relate these to outcome and to identify characteristics associated with greater distress.	IVF	Depression (GHQ)	At the start of the treatment cycle (T1) at treatment day 8 (T2) and following outcome (T3)	At post-treatment, the IVF women show significantly higher scores on depression (t = 2.7; df = 9; *p* < 0.05) in comparison with control group. At other time points, the differences are not significant.
Shih et al. (71)	Taiwan	Longitudinal study	257 (163 pregnancies after IVF, 94 spontaneously conceived pregnancies)	To examine the level of psychological stress experienced by two groups of pregnant women (spontaneous pregnancy, pregnancy after IVF) during their first 20 weeks of pregnancy.	IVF	Stress (PSRS)	9th gestational week (T1), 12th gestational week (T2), and 20th gestational week (T3)	The psychological stress in the 20th gestational week was higher than that in the 12th week, which was higher than that in the ninth week (F = 6.06, *p* < .01). The method of becoming pregnant had no significant influence on pregnancy stress during the first 20 weeks of pregnancy (*p* < .05).
Slade et al. (50)	United Kingdom	Longitudinal study	144	To describe the emotional and relationship characteristics of an unselected sample at the beginning of IVF treatment; to compare, at intake and at 6 months follow-up, those women who became pregnant and those who had completed three cycles of treatment unsuccessfully; and to chart the emotional experiences of the unsuccessful women over the three cycles of treatment.	IVF	Depression (BDI), anxiety (STAI)	In the week before oocyte retrieval (T1), at 4–6 weeks after embryo replacement (T2), at 6 months after either becoming pregnant or being discharged from the programme following the completion of three cycles without a continuing pregnancy (T3)	Comparing these data with the norms for anxiety in working adults indicated that IVF women scored significantly above this comparison group. BDI was significantly higher than controls. Changes in BDI were not significant within cycles of treatment. At follow-up, women in the unsuccessful group showed significantly higher levels of anxiety and depressive symptoms. However, it must be noted that at intake 26% of the women who subsequently became pregnant and 21% of the unsuccessful group were in the mildly depressed category, with a further 7% falling within the moderately depressed range on the BDI for both groups.
Stevenson et al. (68)	**USA**	**Cohort study**	**48 (22 IVF pregnancies, 26 spontaneously conceived pregnancies)**	**To determine the feasibility of recruitment and explore whether women and their partners who conceive *via* IVF experience greater levels of stress and anxiety during pregnancy compared to each other and compared to couples who conceive spontaneously.**	**IVF**	**Stress (PSS), anxiety (STAI)**	**Between 7 and 12 weeks of gestation (T1), between 14 and 20 weeks of gestation (T2), and between 26 and 36 weeks gestation (T3)**	**For the S-Anxiety (STAI) total scores, we found significant sex by trimester (F** **=** **6.17, *p*** **=** **.014) effects on the adjusted mean anxiety scores regardless of group. The interaction effect was caused by the gradual reduction in adjusted mean anxiety across trimesters for women; the anxiety levels of their partners gradually increased over time. No significant differences in stress. No significant main or interaction effects of group were demonstrated for any of the three dependent variables. Furthermore, other main or interaction effect terms in the model were not statistically significant for the PSS total scores.**
Turner et al. (56)	USA	Cohort study	44	To describe stress and anxiety levels over three time points during the IVF cycle, with an interest in documenting the general pattern of stress across the treatment cycle, rather than stress related to a specific procedure.	IVF	Stress (PSS), anxiety (STAI)	Prior to ovarian stimulation (T1), one day prior to oocyte retrieval (T2), and 5–7 days post embryo transfer (T3)	For the STAI State, STAI Trait, and PSS values, there was no main effect of time, no main effect of patient status, and no interaction between time and patient status. Using logistic regression models to predict pregnancy, we found that all scores at T2 were a significant predictor of pregnancy. Mean STAI-State scores were significantly elevated over the normative population mean of 35.20 at all three time points (all *p* values 0.01).
Udry-Jørgensen et al. (47)	NI	Cohort study	96 (47 IVF/ICSI pregnancies, 49 spontaneously conceived pregnancies)	The first aim of the study was to understand the changes in the psychological status of the parents to be from before to after the first-trimester prenatal screening test at around 12 weeks of gestational age, by comparing state anxiety, prenatal attachment, and prepartum depression in couples from an SC group with couples who had undergone *in vitro* fertilization or intracytoplasmic sperm injection.	IVF + ICSI	Anxiety (STAI, cutoff 40)	Around 12th gestational week	Ten (19%) IVF women and eight (16%) spontaneous conception women scored above cutoff for clinical anxiety.
Verhaak et al. (36)	Netherlands	Longitudinal study	207	To determine differences in emotional status (anxiety and depression) and marital satisfaction in pregnant and nonpregnant women before and after their first cycle of IVF and ICSI.	IVF + ICSI	Depression (BDI-PC, cutoff 4), anxiety (STAI)	Before the start of medication, 3 to 10 days before the beginning of the first cycle of IVF and ICSI (T1), 3 to 4 weeks after the pregnancy test after the first cycle (T2) and, in the event of pregnancy, after the first transvaginal ultrasound.	Eight % of women had BDI-PC score higher than cutoff at T1. There was no deviation in state anxiety scores measured with the State and Trait Anxiety Inventory from those of an age-matched and sex-matched normal group at T1. After the first treatment cycle, the number of women who scored above the four-point cutoff for clinically relevant types of depression increased by 60% in the nonpregnant group and remained stable in the pregnant group. Analysis of univariate effects revealed an effect of time on depression PC (F (1, 112 g) = 12.18; *P* = .00)
Verhaak et al. (51)	Netherlands	Longitudinal study	148	To examine the emotional response to IVF from pre-treatment to 6 months post-treatment and factors that contributed to that course.	IVF + ICSI	Depression (BDI-PC, cutoff 4), anxiety (STAI cutoff 48)	Five-10 days before, assessments made (T1) just after the final cycle (4 weeks after pregnancy test) (T2) and follow-up assessments 6 months after the last cycle (T3)	The results of the MANOVA for women did not reveal any significant effect for time for either anxiety or depression. However, a significant interaction effect for time X treatment outcome was indicated for anxiety [F (2,146) = 6.5; *P* < 0.01] and for depression [F (2,146) = 12.9; *P* < 0.01]. *post hoc* t-tests for non-pregnant women revealed a significant increase in both anxiety [t(1,64) = −2.5; *P* = 0.02) and depression [t(1,64) = −2.9; *P* = 0.01] between T1 and T2, whereas pregnant women showed a decrease in anxiety [t(1,82) = 3.2; *P* = 0.00] and depression [t(1,82) = 3.4; *P* = 0.00] in the same period. *post hoc* t-tests did not reveal any change in anxiety [t(1,64) = −0.74; *P* = 0.46) or depression [t(1,64) = 0.18; *P* = 0.86] between T2 and T3 in both pregnant and non-pregnant women. Of the women in the unsuccessful group 23% at T2 and 20% at T3 scored above the threshold scores for subclinically relevant forms of anxiety, for depression: 20% at T2 and 25% at T3.
Verhaak et al. (37)	Netherlands	Longitudinal study	107	To gain more insight into long-term psychological adjustment to IVF in women.	IVF + ICSI	Depression (BDI-PC, cutoff 4), anxiety (STAI cutoff 48)	Before the start of treatment (T1), after the last treatment cycle (T2) and 6 months after the last cycle (T3)	Significant decrease of anxiety T1–T2 (t = 3.1; *P* = 0.03) in women who gave birth after IVF, significant decrease of depression T1–T2 (t = 2.6; *P* = 0.01) in women who gave birth after IVF. Significant increase of depression T1–T2 (t = 2.1; *P* = 0.05) in women who did not give birth. Significant time effect in depression regardless giving birth (F = 3.2; *P* = 0.02). When only the percentages of the group with clinically relevant forms of depression were considered, at pretreatment (T1) 12% scored above the threshold level; just after the last treatment cycle (T2) 20%; 6 months later (T3) 25%. When clinically relevant forms of anxiety were considered, the percentages were 13% at T1, 23% at T2, 20% at T3.
Vikström et al. (65)	Sweden	Case-control study	12,085 (3,532 women who gave birth after IVF, 8,553 women after spontaneous conception)	To examine whether women who undergo IVF treatment are at greater risk of postnatal suicide or postnatal depression (PND) requiring psychiatric care, compared with women who conceive spontaneously	IVF	Depression (ICD-10 diagnostic codes F32-F39)	0–365 days postpartum	Initial analyses showed that PND was more common in the control group than in the IVF group (0.8 versus 0.4%; *P* = 0.04); however, these differences disappeared when confounding factors were controlled for. A history of any psychiatric illness (*P* = 0.000; odds ratio, OR = 25.5; 95% confidence interval, 95% CI = 11.7–55.5), any previous affective disorder (*P* = 0.000; OR = 26.0; 95% CI = 10.5–64.0), or specifically a personality disorder (*P* = 0.028; OR = 3.8; 95% CI = 1.2–12.7) increased the risk of PND. No woman in either group committed suicide during the first year after childbirth.
Vikström et al. (75)	Sweden	Case-control study	29,036 (10,412 women who gave birth after IVF, 18,624 women after spontaneous conception)	To assess if there is a difference in postpartum psychosis risk between women who give birth after IVF treatment and women who give birth after spontaneous conception.	IVF	Postpartum psychosis (ICD-10 diagnostic codes F20-F31 and F531)	0–365 days postpartum	There were no differences in PPP prevalence between the IVF group and the control group (0.3%, *n* = 29 versus 0.4%, *n* = 77) in the chi-square analysis (*P* = 0.169) or the multiple logistic regression analyses (*P* = 0.646; odds ratio (OR): 1.178; 95% CI: 586–2.365).
Vilska et al. (66)	Finland	Longitudinal study	857 (458 pregnancies after IVF, 399 spontaneously conceived pregnancies)	To evaluate the psychological well-being of ART and spontaneously conceiving parents of twins and singletons.	IVF + ICSI	Depression, anxiety (GHQ-36)	2nd trimester of pregnancy (T1), and when the children were 2 months (T2) and 1-year old (T3).	The IVF/ICSI mothers had lower levels of symptoms of depression than their respective controls during pregnancy (F = 12.09, *P* < 0.001). Differences in other time points were not significant.
Visser et al. (69)	Netherlands	Longitudinal study	126	To examine psychological aspects of IVF.	IVF	Anxiety (STAI)	Before IVF (T1) and around 3 weeks after the planned date of egg collection (T2)	The mean scores of IVF women for the state anxiety were significantly higher than those of a local, but representative, population in the age range 16–40 years and those of students in a “state of rest” (during a course). The women had a significantly lower state anxiety score than those of students in a “state of stress” (prior to sitting examinations) and those of psychiatric patients. No significant difference between the two time points.
Volgsten et al. (38)	Sweden	Cohort study	545	To determine the prevalence of psychiatric disorders in infertile women and men undergoing IVF treatment.	IVF + ICSI	Psychiatric disorders (PRIME-MD)	On the day of oocyte retrieval	Major depression was the most common mood disorder, prevalent in 10.9% of females, 8.5% had minor depression. Any anxiety disorder was encountered in 14.8% of females. Of the 413 women in the study sample, 127 (30.8%) had one or more psychiatric diagnoses.
Wan et al. (42)	China	Cohort study	456	To observe the psychological status and analyze the influencing factors among pregnant women undergoing *in vitro* fertilization	IVF	Depression (EPDS cutoff 13), anxiety (STAI, cutoff 50)	18th-29th gestational week	In this study, 191 (41.89%) patients were diagnosed with anxiety disorder, and 131 (28.73%) patients were diagnosed with depression.
Wu et al. (39)	China	Cohort study	288	To explore the relationship between coping strategies and depression, and the risk factors of depression among Chinese women in infertile couples undergoing IVF.	IVF + ICSI	Depression (CES-D10, cutoff 10)	Between oocyte retrieval and before embryo transfer	The incidence of depression was 22.6%.
Yong et al. (52)	United Kingdom	Longitudinal study	37	To identify the stage/s of IVF treatment where a woman is most vulnerable to psychological stress, and to assess the Mean Affect Adjective Check List (MAACL) as a measure of psychological functioning during IVF treatment.	IVF	Depression, anxiety (MAACL)	Before treatment (T1), before embryo transfer (T2), before pregnancy test (T3)	Apart from anxiety scores for T2, the hostility, depression, and anxiety scores for T3 were significantly higher than the corresponding scores for T1 and T2 (*P* < 0.001). Anxiety scores for T2 and T3 were not significantly different.

BAI, beck anxiety inventory; BDI, beck depression inventory; BDI/SF, beck depression inventory, short form; BDI-PC, beck depression inventory, primary care; BSRS-5, brief symptom rating scale; CES-D10, center for epidemiologic studies short depression scale; DACL, depression adjective checklist; EPDS, Edinburgh postpartum depression scale; GHQ, general health questionnaire; HADS, Hospital anxiety and depression scale; HAM-A, Hamilton anxiety rating scale; ICD-10, International classification of diseases, 10th revision; MAACL, mean affect adjective check list; PRIME-MD, primary care evaluation of mental disorders; PSRS, pregnancy stress rating scale; PSS, perceived stress scale; QIDS, quick inventory of depressive symptomatology; SAS, Zung self-rating anxiety scale; SDS, Zung self-rating depression scale; STAI, state-trait anxiety inventory; studies in **bold** are included in the meta-analysis.

The most common tools to measure symptoms of depression were the Beck Depression Inventory (BDI) and the Edinburgh Postpartum Depression Scale (EPDS). Both were used in six studies (21%). Most of the studies concerning anxiety (*n* = 20.74%) used the State-Trait Anxiety Inventory (STAI). Thirty studies (68%) reported that the intervention was IVF, and in 14 studies (32%) the intervention was a combination of IVF and ICSI.

### Quality assessment

Table 2 shows the results of ROBINS-I. Most of the studies (*n* = 30.68%) received a *critical* risk of bias rating in at least one category, thus the overall rating is *critical* (29). To show the difference between the studies, we only present ratings in each domain. All studies in the meta-analysis received a *critical* rating in the selection category, as they included only women who conceived after IVF and omitted those who did not.

**Table 2:** Quality assessment – risk of bias in Non-randomized studies of intervention (ROBINS-I).

Study	Confounding	Selection	Measurement of interventions	Deviations from intended interventions	Missing data	Measurement of outcomes	Selection of the reported result
Arvanitidou et al. (40)	**Moderate**	**Critical**	**Low**	**Low**	**Low**	**Low**	**Low**
Awtani et al. (53)	Serious	Low	Low	Serious	Serious	Moderate	Low
Cozzolino et al. (57)	Moderate	Critical	Low	Low	Low	Low	Low
Dayan et al. (72)	Serious	Critical	Low	Low	Moderate	Low	Low
Gabnai-Nagy et al. (58)	Critical	Low	Low	Low	Serious	Moderate	Low
Gambadauro et al. (41)	**Moderate**	**Critical**	**Low**	**Low**	**Moderate**	**Low**	**Low**
García-Blanco et al. (59)	**Moderate**	**Critical**	**Low**	**Low**	**Low**	**Low**	**Low**
Globevnik Velikonja et al. (60)	Serious	Critical	Low	Low	Moderate	Low	Low
Harf-Kashdaei & Kaitz (61)	**Moderate**	**Critical**	**Low**	**Low**	**Low**	**Low**	**Low**
Harlow et al. (54)	Serious	Low	Low	Low	Low	Low	Low
Hashemieh et al. (46)	Moderate	Critical	Low	Low	Low	Low	Low
Jongbloed-Pereboom et al. (73)	Moderate	Critical	Low	Low	Moderate	Low	Low
Kato et al. (32)	Serious	Low	Low	Low	Moderate	Moderate	Low
Klock & Greenfeld (62)	**Serious**	**Critical**	**Low**	**Low**	**Moderate**	**Low**	**Low**
Kong et al. (33)	Moderate	Low	Low	Low	Serious	Low	Low
Lee et al. (43)	Moderate	Critical	Low	Low	Serious	Moderate	Low
Li et al. (44)	Moderate	Critical	Low	Low	Low	Moderate	Low
Lin et al. (70)	Critical	Low	Low	Low	Low	Moderate	Low
Liu et al. (34)	Low	Low	Low	Low	Low	Moderate	Low
Lukse & Vacc (35)	Moderate	Low	Low	Low	Serious	Moderate	Low
Mahajan et al. (55)	Low	Low	Low	Low	Serious	Moderate	Low
Massarotti et al. (48)	Low	Low	Low	Low	Low	Moderate	Low
McMahon et al. (67)	**Low**	**Critical**	**Low**	**Low**	**Moderate**	**Low**	**Low**
McMahon et al. (63)	Moderate	Critical	Low	Low	Low	Low	Low
Merari et al. (49)	Critical	Low	Low	Low	Moderate	Moderate	Low
Munk-Olsen & Agerbo (74)	Low	Low	Low	Low	Low	Low	Low
Raoul-Duval et al. (45)	Serious	Critical	Low	Low	Serious	Low	Low
Reading et al. (64)	Critical	Low	Low	Low	Low	Low	Low
Shih et al. (71)	Low	Critical	Low	Low	Moderate	Low	Low
Slade et al. (50)	Critical	Low	Low	Low	Serious	Moderate	Low
Stevenson et al. (68)	**Serious**	**Critical**	**Low**	**Low**	**Serious**	**Low**	**Low**
Turner et al. (56)	Low	Low	Low	Serious	Serious	Moderate	Low
Udry-Jørgensen et al. (47)	Low	Critical	Low	Low	Moderate	Low	Low
Verhaak et al. (36)	Critical	Low	Low	Low	Serious	Moderate	Low
Verhaak et al. (51)	Critical	Low	Low	Low	Serious	Moderate	Low
Verhaak et al. (37)	Critical	Low	Low	Low	Serious	Moderate	Low
Vikström et al. (65)	Moderate	Critical	Low	Low	Moderate	Moderate	Low
Vikström et al. (75)	Moderate	Critical	Low	Low	Low	Moderate	Low
Vilska et al. (66)	Critical	Critical	Low	Low	Serious	Low	Low
Visser et al. (69)	Serious	Low	Low	Low	Serious	Moderate	Low
Volgsten et al. (38)	Serious	Low	Low	Low	Moderate	Moderate	Low
Wan et al. (42)	Low	Critical	Low	Low	Low	Moderate	Low
Wu et al. (39)	Low	Low	Low	Low	Low	Moderate	Low
Yong et al. (52)	Moderate	Low	Low	Low	Low	Moderate	Low

Low risk of bias = the study is comparable to a well-performed randomized trial; Moderate risk of bias = the study appears to provide sound evidence for a non-randomized study but cannot be considered comparable to a well-performed randomized trial; Serious risk of bias = the study has some important problems; Critical risk of bias = the study is too problematic to provide any useful evidence on the effects of intervention (27). Studies in **bold** are included in the meta-analysis.

### Results of included studies

Table 1 shows the results of all studies, which are summarized below according to their focus and study design.

### Studies assessing the prevalence of symptoms of depression

Eight studies (32–39) addressed the prevalence of symptoms of depression at different time points during IVF/ICSI treatment. This prevalence ranged from 7% (34) to 54% (32). Furthermore, six different instruments were used to assess symptoms of depression.

Three studies (40–42) focused on the prevalence of symptoms of depression in pregnancy conceived after IVF. The assessment was conducted at different time points, between the 17th and 32nd gestational weeks. The reported rates ranged from 9% (41) to 29% (42). Two studies used the EPDS with different cutoffs: 12 (41) and 13 (42). The third study (40) used the BDI.

Four studies (41, 43–45) concerned the prevalence of symptoms of depression after childbirth following an IVF/ICSI pregnancy. The assessment was conducted at different time points up to nine months postpartum. The reported rates ranged from 9% (41) to 47% (44). Two studies used EPDS with different cutoffs: 10 (44) and 12 (41). One study (43) used the BDI, and the remaining one (45) used interviews to assess the symptoms of depression.

### Studies assessing the prevalence of symptoms of anxiety

Five studies (32–34, 37, 38) analyzed the prevalence of symptoms of anxiety during IVF/ICSI treatment. These were measured at various time points during the intervention and ranged from 14% to 45%. Two studies (32, 37) used the STAI, two (33, 34) used the Zung Self-Rating Anxiety Scale, one used the PRIME-MD (38), and one (32) did not disclose the cutoff it used.

Four studies (40, 42, 46, 47) focused on the prevalence of symptoms of anxiety in pregnancy conceived after IVF. Two studies (40, 47) found that their prevalence was 19%. Arvanitidou et al. (40) assessed outcomes between the 30th and 32nd gestational weeks using the Hamilton Anxiety Rating Scale (HAM-A); this study did not identify the cutoff used. Udry-Jørgensen et al. (47) conducted their assessment in approximately the 12th gestational week using the STAI with a cutoff of 40. Wan et al. (42) estimated prevalence of symptoms of anxiety between 18th and 29th gestational weeks at 42% using the STAI with a cutoff of 50. Hashemieh et al. (46) found that 32.5% of women had moderate to severe anxiety when measured between the eighth and 42nd gestational weeks using the BAI.

### Longitudinal studies of depression trajectories

Seven studies (35, 37, 48–52) explored the trajectories of the symptoms of depression throughout IVF/ICSI treatment. Two (35, 48) found no significant difference between the measurement time points. Merari et al. (49) found no significant difference between the time points before the treatment and before pregnancy tests. Yong et al. (52) found a significant increase in depressive symptoms between the start of the treatment and the embryo transfer and between the embryo transfer and the pregnancy test.

Verhaak et al. (37, 51) found a significant decrease in levels of depressive symptoms before the treatment and six months after the last cycle but only in women whose treatment was successful. On the contrary, in women who did not give birth after IVF, the levels of symptoms of depression significantly increased. Similarly, Slade et al. (50) observed a significant increase in symptoms of depression in women who did not get pregnant.

### Longitudinal studies of anxiety trajectories

Ten studies (37, 48–56) explored the trajectories of symptoms of anxiety throughout IVF/ICSI treatment. All but one study (56) found significantly different levels of anxiety between the observed time points. Harlow et al. (54), Mahajan et al. (55), and Yong et al. (52) observed a significant increase in anxiety levels between pretreatment and the later stages of treatment. On the contrary, Massarotti et al. (48) found a significant decrease in symptoms of anxiety between the time points.

Verhaak et al. (37, 51) found a significant decrease in symptoms of anxiety between pretreatment and the end of the treatment cycle but only in women who subsequently gave birth. In women who did not conceive, the levels of anxiety increased between these time points. This tendency was also reported by Slade et al. (50). Merari et al. (49) found significant differences in the levels of symptoms of anxiety among the four time points, but the levels did not increase or decrease continuously. Awtani et al. (53) found a significant main effect of time on levels of anxiety, but the only significant difference between two time points was an increase between the day of embryo transfer and 10 days afterward.

### Comparative studies assessing depression

Sixteen studies (33, 40, 41, 45, 49, 50, 57–66) compared symptoms of depression throughout the IVF/ICSI treatment, resulting pregnancy, and postpartum periods with control groups or norms. Four (33, 49, 58, 64) assessed symptoms of depression during various phases of the IVF treatment. Two (33, 49) observed higher symptoms of depression in the IVF group when compared to controls, although Merari et al. (49) found an exception at one time point of measurement (the morning before embryo transfer). On the contrary, Gabnai-Nagy et al. (58) observed lower symptoms of depression in the IVF group. Reading et al. (64) found higher symptoms of depression in the IVF group compared to controls only post-treatment but found no significant differences in the beginning or on day eight of the treatment. Furthermore, Slade et al. (50) observed significantly higher symptoms of depression in IVF women compared to adult norms.

Five studies (40, 41, 59, 61, 62) compared symptoms of depression in the third trimester of pregnancy conceived by IVF with those in a control group of spontaneously pregnant women. Our meta-analysis describes them in more detail. Two other studies focused on symptoms of depression in pregnancy. Vilska et al. (66) observed lower symptoms of depression in the second trimester of pregnancy in the IVF/ICSI group. Globevnik Velikonja et al. (60) found no significant difference between pregnant women who conceived by IVF and those who conceived spontaneously in terms of either the levels or the incidence of depression.

Six studies (41, 45, 57, 63, 65, 66) compared symptoms of depression in IVF and control groups at various time points in the postpartum period. Only one study (65) found significantly more women with a diagnosis of depression in the control group. These differences disappeared, however, when controlling for confounders.

### Comparative studies assessing anxiety

Fifteen studies (33, 40, 49, 50, 54, 56, 58–63, 67–69) compared symptoms of anxiety at various time points of IVF/ICSI treatment and resulting pregnancy with control groups or norms. Seven assessed the symptoms of anxiety at a minimum of one time point throughout the IVF treatment. Five (33, 49, 50, 54, 56) found higher levels of anxiety in the IVF groups. The remaining two studies (58, 69) found higher levels of symptoms of anxiety in the control groups. Furthermore, Harlow et al. (54) found higher levels of anxiety in a group undergoing IVF with hormonal stimulation compared to a group without stimulation.

Six studies (40, 59, 61, 62, 67, 68) assessed anxiety symptoms in the third trimester of pregnancy; the meta-analysis below describes these in more detail. The study by Globevnik Velikonja et al. (60) was the only other study measuring anxiety in pregnancy, but its time range was too wide to be included in the meta-analysis. Nevertheless, this study found no significant differences between the IVF and control groups. Only McMahon et al. (63) assessed levels of anxiety postpartum. They found no significant difference between groups at either four or 12 months postpartum.

### Studies assessing stress

Five studies (53, 56, 68, 70, 71) assessed differences between stress levels at various time points of the treatment and pregnancy. Three studies (53, 56, 70) focused on the treatment period. While Lin et al. (70) observed significantly more participants experiencing distress during embryo transfer than during oocyte pickup, Awtani et al. (53) reported that, on the day of embryo transfer, women felt less stressed than at the beginning of the treatment. Turner et al. (56) found no significant difference between three time points of assessment. Both Shih et al. (71) and Stevenson et al. (68) found no significant differences in stress levels between pregnant women who conceived after IVF and those who conceived spontaneously. Shih et al. (71), however, found that stress levels increased throughout pregnancy.

### Studies assessing other mental health outcomes

Four studies (72–75) focused on mental-health outcomes other than depression, anxiety, or stress. A Canadian register study by Dayan et al. (72) addressed the risk of receiving any psychiatric diagnosis within one year postpartum. Compared to women who conceived spontaneously, women undergoing IVF had a lower crude absolute risk but a higher adjusted relative risk of mental illness. Furthermore, 3.5 per 1,000 postpartum women who conceived by IVF had mood or anxiety diagnoses. In a Danish register study, Munk-Olsen and Agerbo (74) estimated the incidence of any mental-health disorder within 90 days postpartum in women who conceived after IVF at 11.3 per 1,000 compared to 3.6 per 1,000 within 90–365 days postpartum. In women who did not give birth following IVF, the incidence was 3.8 per 1,000.

Jongbloed-Pereboom et al. (73) compared the common mental-health problems (defined as >80th of the General Health Questionnaire) of women within one year of giving birth following IVF with women who conceived spontaneously and found no significant difference. Vikström et al. (75) found no significant difference when comparing the incidence of postpartum psychosis in women who gave birth after IVF and those who had a spontaneous pregnancy.

## Meta-Analyses

### Meta-Analysis on the effect of *in vitro* fertilization on symptoms of depression

Five studies were included in the meta-analysis on the effect of IVF on symptoms of depression, with 340 participants in IVF groups and 3,503 in control groups. A Q-test showed a moderate but not significant heterogeneity in the included studies (*Q* = 5.97, *p* = .20, tau² = .01, *I²* = 36.91%). Rosenthal's failsafe *N* showed a potential risk of publication bias (*N* = 4, *p* = .018), which was not detected by the Begg and Mazumdar rank correlation test (*p* = 1.0) or Egger's regression (*p* = 0.91).

**Figure 2:**
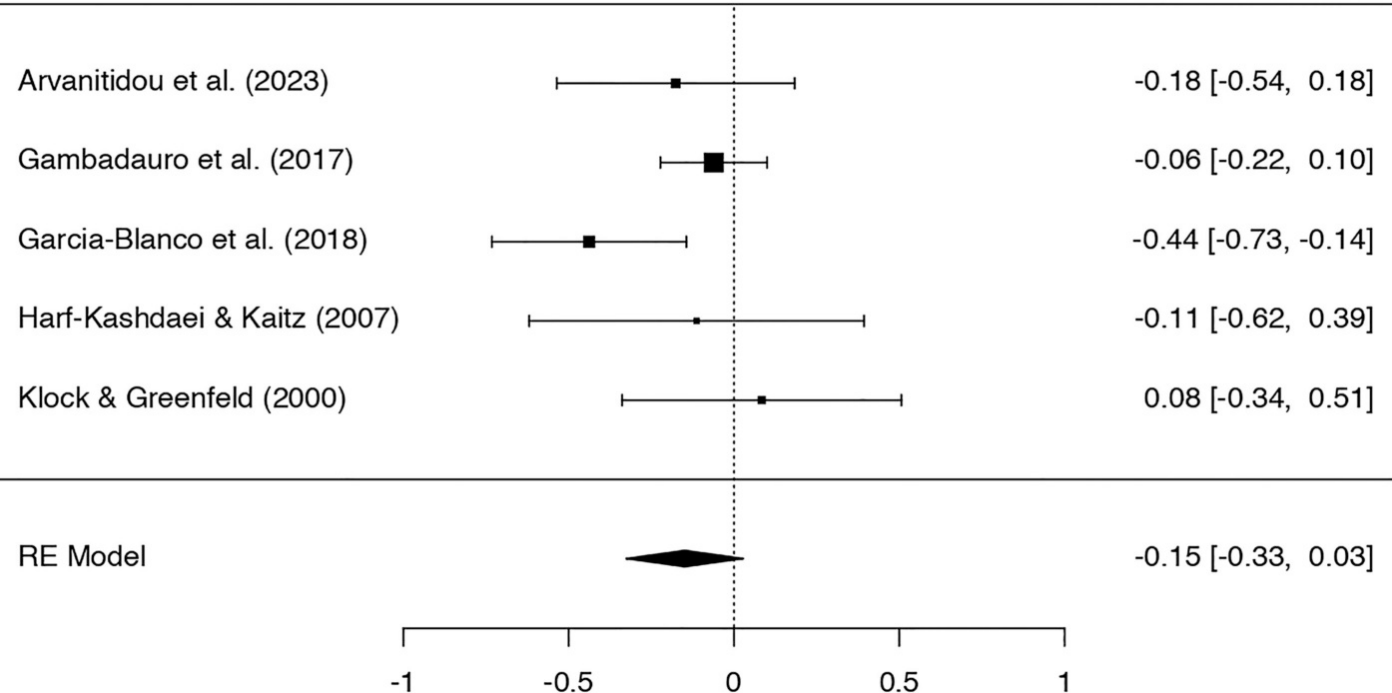
Forest plot of studies on symptoms of depression.

The standardized mean difference (*SMD*) ranged from −.44 to.08; the average *SMD* based on the random-effects model was −.15; 95% *CI* [−.33,.03]. Thus, women in IVF groups had lower symptoms of depression than those in the control groups, but the average *SMD* did not differ significantly from zero (*Z* = −1.65, *p* = .10). See Figure 2 for the forest plot.

### Meta analysis on the effect of *in vitro* fertilization on symptoms of anxiety

Six studies were included in the meta-analysis on the effect of IVF on symptoms of anxiety, with 280 participants in the IVF groups and 744 in the control groups. A Q-test showed no significant heterogeneity in the included studies (*Q* = 3.97, *p* = .55, tau² = .00, *I²* = .00%). Rosenthal's fail-safe *N* showed a potential risk of publication bias (*N* = 31, *p* < .001), which was not confirmed by the Begg and Mazumdar rank correlation test (*p* = 0.72) or Egger's regression (*p* = 0.73).

**Figure 3:**
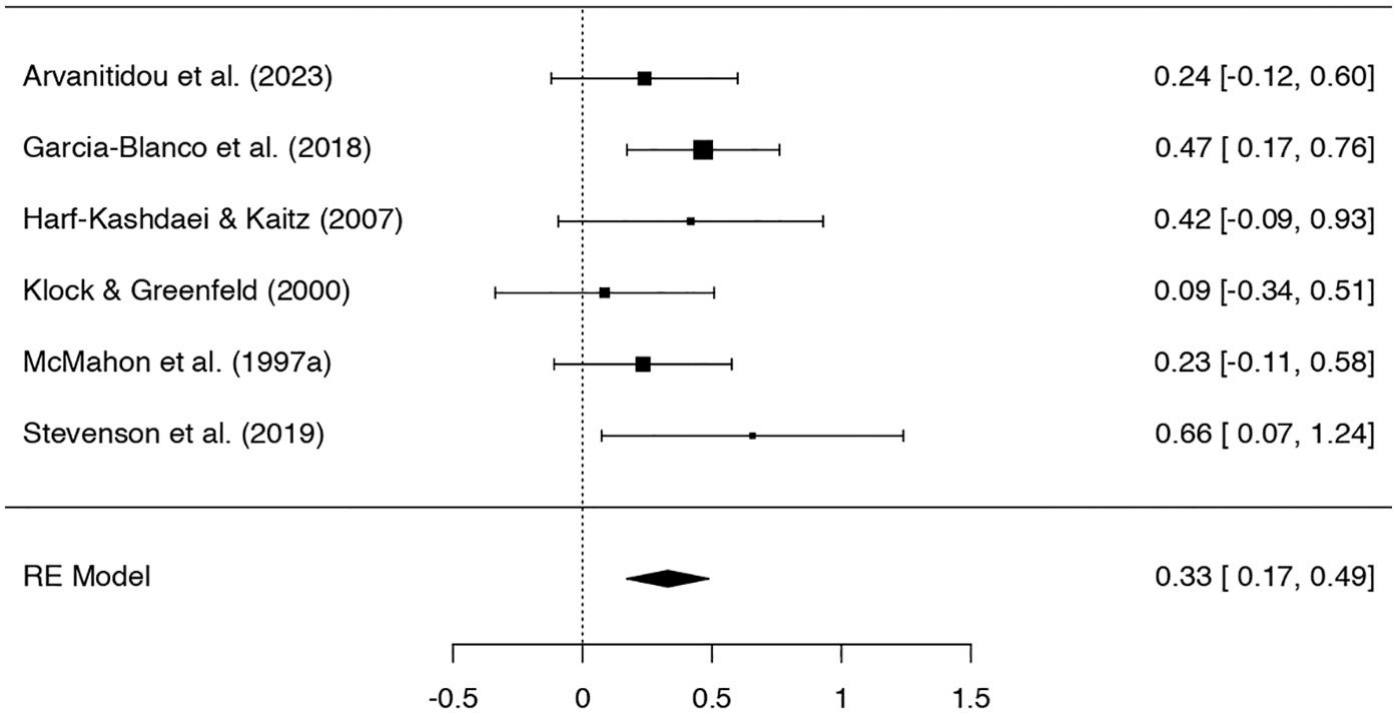
Forest plot of studies on symptoms of anxiety.

The *SMD* ranged from.09 to.66; the average *SMD* based on the random-effects model was.33; 95% *CI* [.17,.49]. Thus, women in the IVF groups showed significantly higher symptoms of anxiety than those in the control groups (*Z* = 4.09, *p* < .001). See Figure 3 for the forest plot.

## Discussion

In this systematic review and meta-analysis, we assembled empirical evidence about effects of *in vitro* fertilization (IVF) or intracytoplasmic sperm injection (ICSI) treatment on women's mental health in the perinatal period. We included 44 studies that covered 858,966 participants and were conducted in 20 countries. The substantial number of studies using various study designs, together with the broad inclusion criteria, have contributed to the notable heterogeneity of the results.

Most frequently, the included studies assessed symptoms or diagnoses of depression as an outcome measure. Estimates of the prevalence of depression throughout the treatment and perinatal period varied substantially, ranging from 7% to 54% compared to general estimates of 15% in pregnancy (15) and 14% postpartum (16). The prevalence of anxiety during the treatment and pregnancy ranged from 14% to 45% compared to 15% in the general population of pregnant women (17).

One reason for the evident discrepancy between included studies might be the use of various assessment tools and different cutoffs, such as 10 (44), 12 (41) and 13 (42) in studies using the EPDS and 40 (47), 48 (37), and 50 (42) in studies using the STAI. Furthermore, the rates of incidence of any psychiatric disorder, defined by diagnostic codes in two register studies, were very low: according to Dayan et al. (72), 3.5 per 1,000 postpartum women experienced a new onset of mood or anxiety disorder after IVF; Munk-Olsen and Agerbo (74) estimated the incidence of any new psychiatric disorder at 3.8 per 1,000 postpartum women after IVF. Although the outcomes of these studies focusing on incidence are not fully comparable with those of the studies assessing prevalence, it can be assumed that the rates of mental difficulties are higher when measured by self-reported questionnaires than the rates of psychiatric diagnoses. This implies the need to interpret the results of this systematic review and meta-analysis in terms of the *symptoms* of mental disorders, not mental disorders *per se*.

The alarmingly high prevalence of depressive symptoms found in some studies were surprising in the context of comparative studies, most of which found no significant differences between women conceiving after IVF and in the control groups. During the treatment, the results of studies were heterogeneous, some indicating significant differences between the two groups. These differences were observed in both directions, however, making it challenging to draw clear conclusions. No statistically significant mean difference in the third trimester was confirmed by the meta-analytical part of this study. This is in line with a meta-analysis by Chen et al. (24) with a broader focus on the whole perinatal period and another by Gressier et al. (76) analyzing depression in the postpartum period.

Studies comparing the symptoms of anxiety in IVF/ICSI and control groups yield more consistent results: most have found higher levels of anxiety in the IVF/ICSI groups. This effect was also confirmed by the meta-analytical part of this study, although the standardized mean difference is rather low. According to Schaller et al. (77), the major stressors leading to anxiety in women undergoing IVF are fear of obtaining a negative pregnancy test, disappointment after anticipation of pregnancy, and childlessness at an older age.

The results of studies assessing the longitudinal trajectories of the symptoms of depression, anxiety, and stress were also very heterogeneous. Notably, studies that separately compared the levels of symptoms in women who conceived after IVF/ICSI and women who did not found a significant decrease of symptoms in the successful group and a significant increase in the unsuccessful group between the time point before the treatment and during pregnancy (37, 50, 51). The reason for such an effect might be quite straightforward: couples dealing with fertility problems are often affected by psychological strain, which might lessen when pregnancy is conceived and persist or deepen when the treatment is unsuccessful. It has been proven that planned pregnancy is a protective factor in developing perinatal depression (15).

A contributing factor to the discrepancies between the results of the mentioned studies might be the difference in the social context of ART in different countries. Kato et al. (32) argue that the high price of the treatment (up to US$6,700) together with difficulties coordinating the treatment with long work hours in a corporate climate in Japan might be a stressor that contributes to mental difficulties in treated women. Furthermore, the social pressure to have children and the persistent preference for male offspring in some Asian countries might also augment the susceptibility to psychopathology. Higher levels of depressive symptoms in the perinatal period in Asian countries were also reported by a meta-analysis by Chen et al. (24). On the contrary, in Sweden, where up to three IVF treatment cycles are funded by regional health services, the social stigma of ART subsides, and the patient-friendliness of the whole treatment process increases (41).

In many countries, IVF/ICSI treatment is expensive and is paid by the patients (78–80). It can be assumed that the treatment might not be accessible to infertile patients with low socioeconomic status, which might contribute to the overrepresentation of participants with higher socioeconomic status in the studies included in this review. It is well known that low socioeconomic status is a risk factor for depressive symptoms in the perinatal period (76), which might also lessen depressive symptoms in IVF women in the included studies.

It is also important to interpret our findings within the broader context of empirical evidence on the psychotropic consequences of women's hormonal environments and other interventions that alter them. Hormonal fluctuations across the menstrual cycle, pregnancy, the postpartum period, and during menopause are known to influence women's well-being and vulnerability to mood disturbances, although the specific effects of these hormones are not clear (81–83). Likewise, exposure to exogenous reproductive hormones through various medical treatments has been associated with changes in mental health (84, 85). Considering the effects of IVF procedures in this context would provide valuable insight into the complex interplay between hormonal environments and women's psychological outcomes.

### Strengths and limitations

In terms of the number of included studies, this study was larger than other systematic reviews and meta-analyses on similar topics (24, 25, 76), resulting in a broader scope of evidence. On the other hand, as we focused solely on the effects of IVF/ICSI and not ART more generally, our study provides more precise information on this kind of treatment. To our knowledge, the meta-analysis concerning symptoms of anxiety is the first of its kind.

A major limitation of the studies included in this systematic review and meta-analysis is that they almost completely omitted women whose treatment was unsuccessful. As indicated in studies by Slade et al. (50), Verhaak et al. (51), and Verhaak et al. (37), this experience might have adverse effect on female patients’ mental health. Thus, the generalizability of our results is strongly limited by this exclusion and very likely underestimates the effects of IVF/ICSI on perinatal mental health.

A significant bias might have been caused by the way confounding factors were approached in the included studies. In some, confounding factors were controlled for well, while other studies did not use proper controlling techniques or failed to control for the most important variables known to be associated with perinatal mental difficulties, such as a history of psychopathology, low income, or social support. Similarly, in the comparative studies included in our meta-analysis, not all control groups were matched according to possible confounders.

Some of the limitations are also reflected in the risk of bias assessment: most of the included studies (*n* = 30.68%) received a *critical* rating in at least one category, implying that the overall risk of bias in these studies is critical. Most frequently, the category of selection of the participants was rated as *critical*, a categorization linked to the noted omission of women who did not get pregnant after IVF/ICSI.

Further limitations should be considered when interpreting the results of both meta-analyses. The studies were fairly homogeneous, but their number was rather small, as were, for the most part, the sample sizes. Most failed to disclose the results of the power analysis indicating the number of participants needed for robust results, and, in some, the sample size was so small that it could have led to biased outcomes. Furthermore, it is likely that the distributions of data in the individual studies were skewed, which can lead to inaccurate outcomes in the meta-analysis. This can be improved by the transformation of the individual participants’ data, which we were unable to access; thus, we consider this a possible limitation. Similar methodological challenges to those described above have been found in previous reviews and meta-analyses of reproductive outcomes after ART, particularly regarding study designs, outcomes, and confounding measures (86, 87).

## Conclusion

The studies included in this systematic review and meta-analysis show considerable heterogeneity in terms of their study designs and the time points of their data collection, and they reached very heterogeneous results. We found, however, significantly higher levels of anxiety in women in the third trimester of pregnancy conceived by IVF compared to women who conceived spontaneously. On the other hand, the standardized mean difference is rather low, and, considering the limitations of the included studies, this result should be interpreted cautiously. Our meta-analysis focused on symptoms of depression only in the third trimester of pregnancy. It would be valuable to conduct such analyses at different time points throughout the perinatal period; the limited number of studies per subgroup, however, would prevent us from drawing meaningful conclusions. Some of the studies assessing the prevalence of symptoms of mental disorders in the perinatal period after IVF/ICSI found alarming results, with this prevalence found to be as high as 54%. This implies the need for increased awareness of possible difficulties among medical staff providing ART. Ideally, mental-health screening with subsequent care by mental-health professionals should be a standard service of fertility clinics.

To reach robust results, future studies should engage larger samples of participants when comparing mental difficulties between women pregnant after IVF and those pregnant spontaneously. To obtain unbiased results, women whose treatment was unsuccessful should not be excluded from the studies. It is crucial for future studies to use standardized, validated tools to provide valid results. There is also a lack of studies concerning such comparison in women in the first two trimesters of pregnancy and postpartum: notably, only one study focusing on symptoms of anxiety in the postpartum period was identified. Future research should also focus on factors contributing to the prevalence of symptoms of depression and anxiety in this population, as the rates vary substantially among the published studies. If these factors are known, it will be easier to predict and prevent mental difficulties in this population.

## Data Availability

The original contributions presented in the study are included in the article/Supplementary Material, further inquiries can be directed to the corresponding author.
